# A Study Protocol for a Parallel-Designed Trial Evaluating the Impact of Plant-Based Diets in Comparison to Animal-Based Diets on Health Status and Prevention of Non-communicable Diseases—The Nutritional Evaluation (NuEva) Study

**DOI:** 10.3389/fnut.2020.608854

**Published:** 2021-02-02

**Authors:** Christine Dawczynski

**Affiliations:** ^1^Junior Research Group Nutritional Concepts, Institute of Nutritional Science, Friedrich Schiller University Jena, Jena, Germany; ^2^Competence Cluster for Nutrition and Cardiovascular Health (nutriCARD), Halle-Jena-Leipzig, Leipzig, Germany

**Keywords:** nutrients, vegetarians, vegans, western diet, nutritional concepts

## Abstract

**Background and Aims:** Currently, there is a continuing upward trend for plant-based lifestyles in Germany and Europe. The implementation of vegetarian and vegan lifestyles is characterized by omitting defined food groups such as fish, meat, sausage (vegetarians), or dairy products and honey (vegans). This carries the risk of an undersupply of valuable nutrients. The NuEva study is designed to examine this hypothesis and to evaluate the impact of plant-based diets on health status and disease risk.

**Methods:** The NuEva study is a parallel-designed trial with at least 55 participants for each diet (vegetarian, vegan, flexitarian [rare meat/sausage consumption, once or twice per week]), and participants who consume a traditional Western diet as the control group. In the screening period critical nutrients are identified for the studied diets by analysis of a broad spectrum of nutrients in the human samples (fatty acids, vitamins, minerals, trace elements, nutrient metabolites).

**Results:** Based on the data from the screening period, defined menu plans, ensuring an adequate nutrient intake in accordance with the nutritional guidelines are prepared for each group. The plans are adapted and personalized to individual energy requirements based on the basal metabolic rate and physical activity level. The compliance with the NuEva concept and their impact on nutrient status and cardiovascular risk factors are validated during the intervention period of the NuEva study over 1 year. To investigate the impact of the studied diets on the microbiome, feces samples are collected at the beginning and after the 12 months intervention period (follow up: 12 months).

**Conclusion:** The NuEva study is designed to investigate the impact of common diets on health and disease status, with focus on prevention of cardiovascular diseases. In addition, the effectiveness of the prepared nutritional coaching strategy, ensuring optimal nutrient intake in accordance with the guidelines, is validated during the intervention period of the NuEva study.

**Clinical Trial Registration:** Registered under ClinicalTrials.gov Identifier no. NCT03582020.

## Importance

Currently, there is a continuing upward trend for vegetarian and vegan lifestyles. The Vegetarierbund Deutschland e.V.^1^ postulates that in 2015, 7.8 million Germans were vegetarians and 900,000 were vegan, whereby about 2,000 vegetarians and 200 vegans join the community in Germany every day. This is also a global trend. It has been estimated, that worldwide, about 1 billion people have adopted a vegetarian-vegan lifestyle.

The implementation of the vegetarian-vegan lifestyle is characterized by omitting defined food groups such as fish, meat, sausage (vegetarians), or additionally dairy products, and honey (vegans). This bears the risk of undersupply of valuable nutrients. Critical aspects of the vegetarian-vegan lifestyle are low intakes of vitamin B12, vitamin D, long-chain omega-3 fatty acids (n-3 LC-PUFA), calcium, iron, zinc, as well as a high phytate intake (1). On the other hand, a vegetarian/vegan diet, usually rich in fruits, vegetables, whole-grains, legumes, nuts, and various soy products, is characterized by a low intake of saturated fat and cholesterol and a high intake of dietary fiber, carotenoids, vitamins and health-promoting phytochemicals (2, 3). Vegan and vegetarian diets are associated with cardiovascular and other health benefits, but little is known about the associations of critical nutrients in plant-based diets and the resulting effects on brain function and mental health.

The Western diet, which is the major nutritional style in Germany, is characterized by high intake of energy, saturated fat, salt, and simple/added sugar and, on the other side, a comparably low intake of vegetables and fruits, resulting in low intake of dietary fibers, PUFA and secondary plant compounds^2^ (4). Obviously, these dietary habits are associated with an increase of cardiovascular risk factors (5, 6).

## Rationale of the NuEva Study

The NuEva study aims to reveal the impact of chosen nutritional habits (Western diet, flexitarians, vegetarians, vegans) on health status and disease risk with respect to physiological benefits or possible pathophysiological consequences, resulting from long-term implementation of the studied diets.

In this context the central issues of the NuEva study are:

- Is it possible to ensure an adequate intake of all essential nutrients?
- Are land-based n-3 PUFA from linseed oil a suitable source to ensure an adequate status of n-3 LC-PUFA?
- What impact has each of the studied diets on state of health and disease and, in particular, on cardiovascular risk?

As the Western diet is the main nutritional style in Germany, it is used as an appropriate comparator for evaluating the physiological impact, resulting from alternative diets, e.g., the vegetarian, vegan and flexitarian diet. There are inherent risks, in both the rising popularity of veganism and vegetarianism and the prevalence of the Western diet because of the likelihood of over- and undersupply of valuable nutrients. This highlights the need of an extensive data collection to serve recommendations, that are based on scientific evidence.

## Methods and Analysis

### Study Design

**Figure 1:**
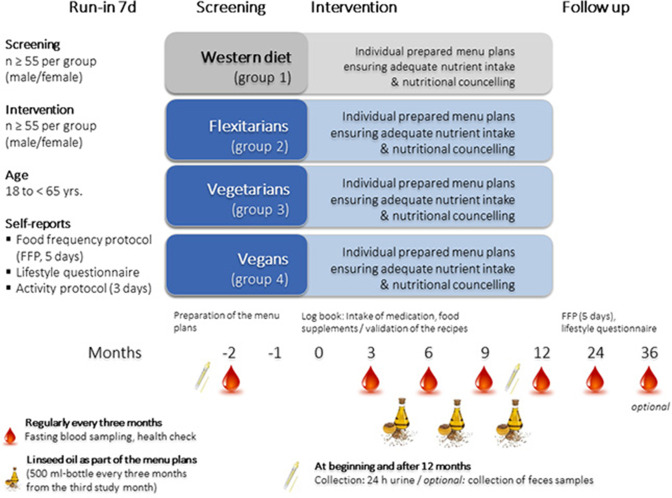
Design of the NuEva study.

For the NuEva study, at least 55 participants for each diet (vegans, vegetarians, flexitarians, and participants who consume a traditional Western diet) are recruited in summer/autumn 2018 according to the following inclusion and exclusion criteria (Table 1). The 2-year trial is carried out in central Germany (recruiting area: central East Germany: Jena-Halle-Leipzig). The allocation ratio is 1:1:1:1. The long-term trial in parallel design consists of four study periods (Figure 1). The Follow-up period ends in December 2020 (optional: winter 2021).

**Table 1:** Inclusion and exclusion criteria in the NuEva study.

**INCLUSION CRITERIA**
▪ Healthy volunteers ▪ Female (pre-menopause) and male gender ▪ Age: 18 to < 65 years ▪ BMI < 30 kg/m^2^ ▪ Adherence to one of the four diets (Western diet, flexitarians, vegetarians, vegans) confirmed by lifestyle and food questionnaires (FFPs over 5d, run-in) ▪ Precondition: stable eating habits for at least 1 year before enrolment
**EXCLUSION CRITERIA**
▪ Patients with any acute or chronic disease (tumor, infection, other), gastrointestinal disease, diabetes mellitus (type I, II), chronic renal disease, diseases of the parathyroid, diseases necessitating regular phlebotomies, or other chronic diseases, which could affect the results of the present study ▪ Use of prescription medicine, which could affect results of the study, including systemic glucocorticoids, and lipid-lowering agents ▪ Dependency on alcohol or drugs ▪ Simultaneous participation in other clinical studies ▪ Inability (physically or psychologically) to comply with the procedures required by the protocol ▪ Changes in eating habits Treatments precluding participation (at least 3 months prior to study start) and/or during the trial ▪ Weight loss or weight gain (> 3 kg) ▪ Fundamental changes in dietary habits (precondition: stable eating habits for at least 1 year before enrolment) ▪ Hormone replacement therapy ▪ Elite athletes (>15 h of strenuous physical activity per week) ▪ Pregnancy or lactation ▪ Transfusion of blood in the last 3 months before blood sample taking

#### Run-in Period—Assessment of Dietary Habits and Lifestyle

To record and document the variety in dietary practices within and among each group, the run-in phase of the NuEva study includes full self-reports of the individual dietary habits using a food frequency protocol (FFP) which had to be maintained over 5 days (Figure 1). The FFP consists of a list of foods that are normally consumed in the Western diet and the corresponding portion information. The participants have to mark the consumption of the listed foods with a line. Food and beverages that are not listed can be added. The FFP based on the template “Freiburger Ernährungsprotokoll” which was provided by the software package PRODI®. The template was adapted on the NuEva study by adding plant-based foods, e.g., tofu, seitan, tempeh, legumes. The daily nutrient intake was calculated by the software package PRODI®. The protocol to record physical activity was adapted to a template which is provided by the German Institute of Nutrition Research (DIfE) (7). NuEva participants kept the activity log for 3 days (pre-condition: 1 day on the weekend). To document medical history, an appropriate questionnaire was developed for the Nueva study. This questionnaire based on a comparable questionnaire available from the LURIC study (8). Available questionnaires from the German National Consumption Survey II (NVS II) and the German health interview and examination survey for adults (DEGS1) are used to consider also the socio-economic status as a confounding factor (9). In detail, the questionnaire include a set of questions about marital status, household size, educational achievement, income, and occupation as well as employment status. To evaluate differences on the occurrence of depressive symptoms between the studied dietary habits, the Patient Health Questionnaire-9 and the Patient Health Questionnaire-15 are used (10, 11).

#### Screening Period—Assessment of Nutrient Status and Health Status

Blood samples, 24 h urine and a fecal sample are collected to examine nutrient status and chosen risk factors for CVD and type-2 diabetes mellitus, parameters reflecting thyroid function and bone health, and inflammatory markers (Figure 1; Table 2). In addition, a health check is conducted, which includes measurement of weight, height, waist circumference, blood pressure, basal metabolic rate and bioelectrical impedance analysis (BIA) according to standard procedures (Table 2).

**Table 2:** Primary and secondary outcome measures in the NuEva study.

***Primary outcome measure*** **(plasma, five times plus follow-up)**
▪ Low density lipoprotein (LDL)/high density lipoprotein (HDL) ratio
***Secondary measures*** **(plasma/serum, five times plus follow-up)**
▪ Total cholesterol, triacylglycerides, lipoprotein (a), apolipoprotein AI, B ▪ Anthropometric and physiological data (height, weight, waist circumference, blood pressure, bioelectric impedance measurement) ▪ Vitamins (A, D, E, B1, B2, B6, folic acid, B12) and vitamin B12 status marker (holotranscobalamin, methylmalonic acid, homocysteine) ▪ Minerals [calcium, sodium, potassium, iron, and iron status markers (ferritin, transferrin)] ▪ Trace elements (copper, zinc, manganese, total selenium and selenoprotein P, glutathione peroxidase (GPx) activity) ▪ Fatty acid distribution in plasma lipids and erythrocyte lipids (> 90 fatty acids) ▪ Additional cardiovascular risk factors (high sensitive c-reactive protein, uric acid) ▪ Diabetes risk factors [fasting glucose, fasting insulin, hemoglobin A1c (HbA1c)] ▪ Clotting markers (alpha prothrombin time, fibrinogen, international normalized ratio, quick value) and blood count ▪ Cholesterol synthesis markers/cholesterol resorption markers ▪ Bone turnover (bone-specific alkaline phosphatase, osteocalcin, β-crosslaps (CTX), N-terminal propeptide of type I procollagen (PINP), parathyroid hormone) ▪ Thyroid function [thyroid-stimulating hormone, free thyroxine (T4), free triiodothyronine (T3)] ▪ Optional: targeted metabolic profiling (AbsoluteIDQ p180 Kit from Biocrates), inclusive unbound free fatty acid profiles in plasma
***Secondary measures*** **(24 h urine, at the beginning and after 12 months)**
▪ Albumin, creatinine ▪ Sodium, magnesium, zinc, selenium, iodine ▪ Heavy metals (arsenic, lead, cadmium, mercury)
***Secondary measures*** **(feces collection, at the beginning and after 12 months)**
▪ Distribution of the gut microbiome

#### Intervention Period—Validation of the NuEva Concept

Based on the screening data and published scientific data, critical nutrients, whose intakes are too low or too high in comparison to the recommendations, are identified. These weak points are addressed by the personalized nutritional concepts which are prepared for each diet. The compliance with the nutritional concepts and its physiological impact on health and disease status are studied by regular analyses of the study parameters (Table 2). Therefore, blood samples are taken every 3 months (five times), and 24 h urine is collected at the beginning and at the end of the intervention period (Figure 1). Fecal samples are also collected at the beginning and after 12 months of the NuEva study to evaluate the impact of the studied diets on the composition of the gut microbiome (Figure 1).

#### Follow-Up

After the implementation of the NuEva concept (intervention period), a 12-months period (or optional 24-months period) without nutritional coaching follows (Figure 1).

### The Nutritional Coaching Concept

**Figure 2:**
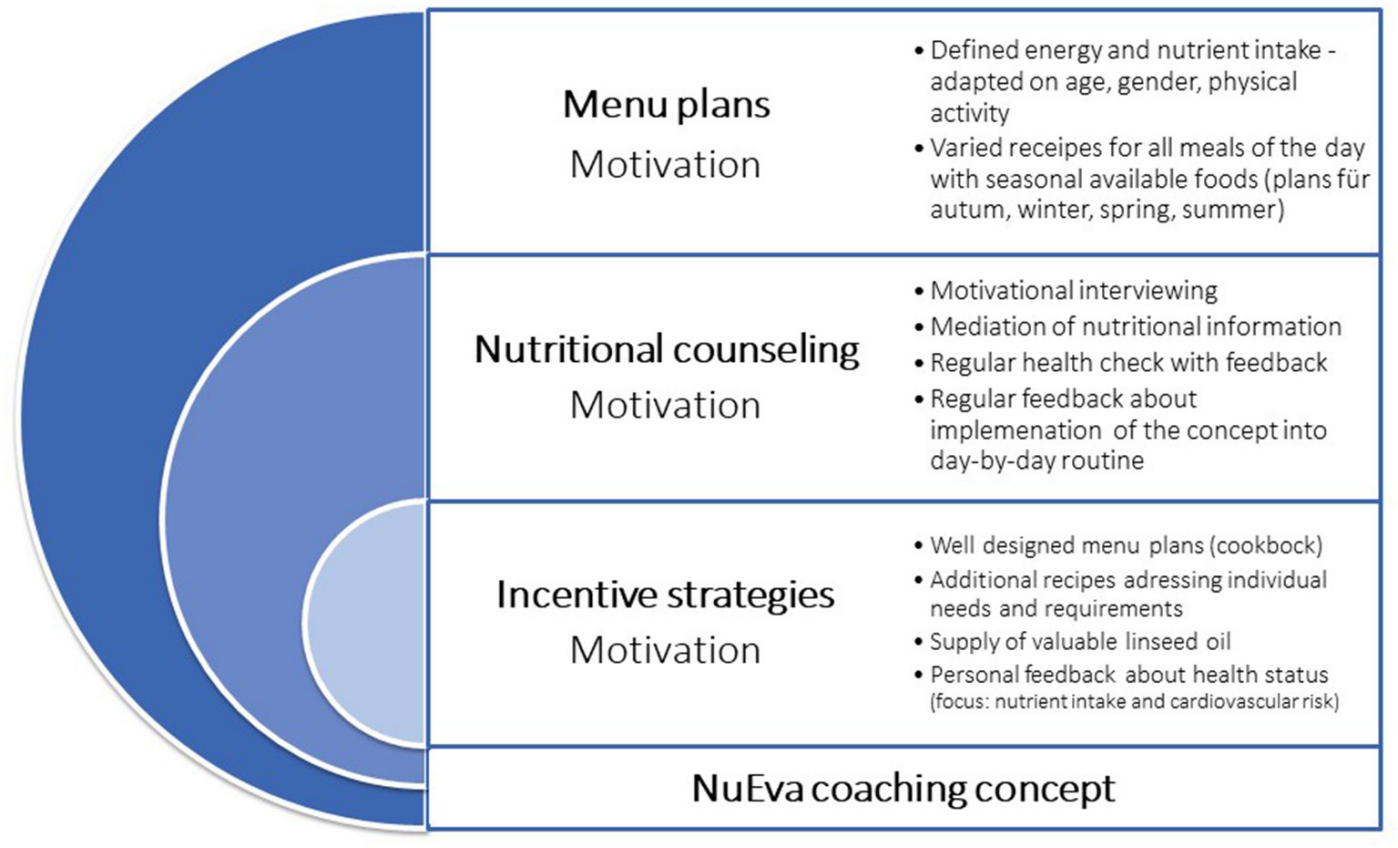
The NuEva coaching concept.

The NuEva coaching concept is developed to improve nutritional behavior based on three columns: (1). personalized menu plans that are adapted to individual needs and requirements, (2). regular nutritional counseling units including feedback, and tracking, and (3). various incentive strategies (Figure 2).

#### Menu Plans

Participants of the NuEva study receive daily menu plans with optimized nutrient profiles over a period of 1 year. In total, participants receive 20 menu plans for each season (autumn, winter, spring, summer) as part of the nutritional counseling units every 12 weeks. The menu plans are validated by regular fasting blood sampling and health checks every 3 months (Figure 1). The regularly analysis of the study parameters, e.g., fatty acid distribution in plasma and erythrocyte lipids allows an evaluation of the compliance with the menu plans as the characteristic fatty acid profiles of the menu plans will be reflected by changes on fatty acid distribution in plasma and erythrocyte lipids (12).

Based on the analysis of the nutrient status in the screening period, critical nutrients are identified for each participant and summarized for each group. Based on this and published scientific data, menu plans and recommendations are prepared for each group and personalized for each participant, ensuring adequate nutrient intake. The plans are adapted to individual energy requirements based on the basal metabolic rate and the physical activity level (PAL).

The menu plans are in accordance with the following hallmarks of the studied diet forms:

Western diet: daily consumption of meat, sausage or fish
Flexitarians: rare consumption of meat, sausage or fish (predominantly high-quality products), ≤ 2 times/week
Ovo-lacto vegetarians: no consumption of meat, sausage or fish
Vegans: no consumption of foods from animal origin

The NuEva menu plans of all four groups are characterized by:

1. Adequate amounts of energy, carbohydrates, protein and fat in accordance with the guidelines of the German Society of Nutrition (13)
2. Defined intake of saturated fatty acids (SFA, < 10% of daily energy), monounsaturated fatty acids (MUFA, > 10% of daily energy), PUFA (~10% of daily energy) and at least two grams of alpha-linolenic acid per day, e.g., through linseed oil (5–10 ml per day between study month 3 and 12; Figure 1).
3. Encouraged consumption of vegetables, fruits, cereals
4. Intake of > 40 g dietary fiber per day based on the MoKaRi concept (14)
5. Salt (maximum of 6 g per day) and sugar reduction (maximum of 50 g per day)
6. Reduced intake of highly processed, calorie-rich, low-nutrient foods (fast foods, convenience products)
7. Optimized intake of vitamins, minerals, and trace elements by commercially available foods, considering seasonal availability of vegetables and fruits.

**Figure 3:**
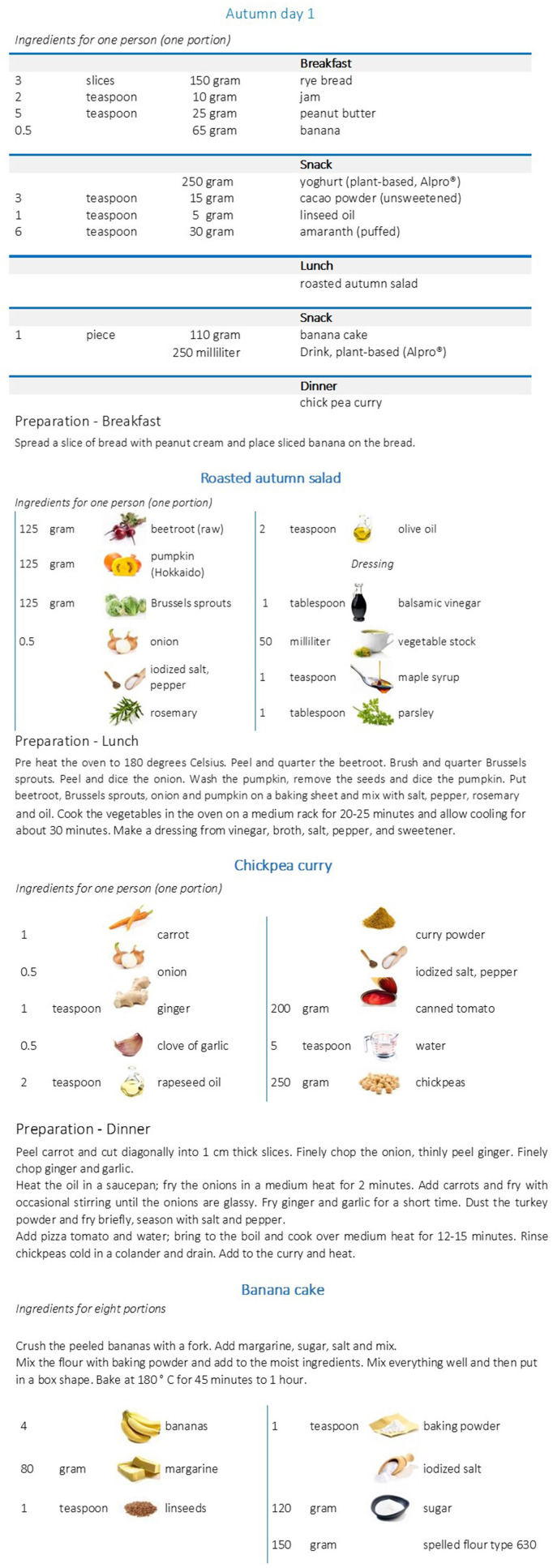
Example for a menu plan (vegan). Menu plan for a woman (25 ≤ 51 years), with a physical activity level (PAL) of 1.6 which corresponds to a daily energy intake of 2,100 kcal (13).

The daily recipes and menu plans are adapted to the individual energy requirement depending on age, gender, basal metabolic rate, and physical activity. Therefore, the menu plans are prepared in eight energy levels, including detailed information about the type and amount of food for breakfast, morning snack, lunch, afternoon snack and dinner. For each meal, a well-designed step-by-step description for preparation is provided (Figure 3).

The software package PRODI® (Nutri-Science, Stuttgart, Germany, version 6.4) for professional dietary counseling and therapy was used to calculate the nutrient content of the menu plans. The recipes for each meal originated from cookbooks. The uniqueness of the menu plans comes from their balanced nutrient profile which considers the individual requirement of each participant. Thus, selected nutrient-rich foods were added to conventional recipes in order to meet the NuEva criteria for daily nutrient intake. For example, fatty cold water fishes are selected to ensure adequate intake of n-3 LC-PUFA, vitamin B12, vitamin D, iodine, and protein for participants who follow the flexitarian diet or the Western diet. Dairy products with moderate fat content are included to help flexitarians, vegetarians and adherents of the Western diet to reach the recommended intake of B vitamins, calcium and other minerals but also high-value protein. Linseed, rapeseed and olive oils, mixed nuts and seeds are added to meet the criteria for fat quality in all diets. Vegetables, chosen fruits, nuts and seeds, cereals, cacao and pulses, such as peas, beans, or lentils are added to ensure optimal intake of vitamins, minerals, trace elements, dietary fibers, protein and secondary plant compounds, e.g., carotenoids and polyphenols (all groups). For vegans special foods, enriched with vitamin B12 and calcium but also the recommendation to supplement vitamin B12 are included to reach the recommended intake of these valuable nutrients. The daily menu plans are provided as well-designed cookbooks with one page for each day on wipeable paper (Figure 3).

#### Lifestyle Coaching—Focus: Nutritional Habits

The regular nutritional counseling units are implemented by using the Motivational Interview technique, based on the transtheoretical model (15–18). In detail, the screening period of the NuEva study starts with participants in the contemplation (“getting ready”) stage. The participants are interested in nutritional facts and wanted to be informed about the pros and cons of their previous nutritional patterns. If they decide to participate in the intervention period, they continue to the next level, called preparation (“ready”) stage. From this point, participants are intending to take action in the immediate future and may begin taking small steps toward changing their nutritional behavior. During the first 6 months of the nutritional coaching program (intervention period), the participants move up to the action level (“current action"). This stage is characterized by changes in their nutritional behavior by implementing the personalized menu plans. In the further course of the intervention period, the participants maintain the learned nutritional behavior by continuous usage of the menu plans (maintenance stage “monitoring”). After the intervention period, participants hopefully get into the termination stage. On this level, subjects will continue the learned healthy eating habits to ensure an adequate nutrient intake, depending on the characteristics of their individual diet.

The nutritional counseling concept includes the following hallmarks (Figure 2):

1. Supply of 80 personalized menu plans, including practical hints to facilitate implementation into the day-to-day routine
2. Providing nutritional facts on the following topics: energy requirement, physiological impact of chosen nutrients (e.g., fats, dietary fibers, sugar, salt) and nutrient-dense foods such as vegetables and fruits, dairy products or plant-based alternatives, and beverages
3. Individual feedback about the course of the study parameters which reflect state of health (Table 2)
4. Defining individual goals depending on the course of the study parameters
5. Exchange about the feasibility of the NuEva concept in the day-by-day routine.

Besides the supply of the personalized menu plans, regular talks and feedback loops are important hallmarks of the NuEva concept (Figure 2). Through them, personal information about life circumstances, individual needs and possible barriers of implementing the NuEva concept are discussed. Based on this, individual solutions to improve compliance are sought. The talks are conducted in a user-friendly environment to improve the well-being of the participants.

### Outcome Measures

The NuEva study aims to investigate the impact of the four studied diets on health status and established risk factors for non-communicable diseases (Table 2). The analyses are conducted every 3 months over 1 year (plus follow-up after further 12 [optional 24] months; Figure 1).

## Statistical Methods

Data management will be conducted using the statistical software system R version 3.5.0. The design allows the comparison of the study outcomes between the studied diets (screening) and the course of the study parameters over the intervention and follow-up periods, respectively. Descriptive statistics will be used to describe each stage of the study (run-in, screening, intervention, and follow-up). Datasets from the screening period will be analyzed using (generalized) linear models, e.g., analysis of covariance (ANCOVA). The endpoints will be adjusted for potential confounding factors, including, but not limited to, gender, age, and BMI. The datasets from the intervention and the follow-up periods will be tested using a repeated measurement analysis with a significance level of 5%. Previously, data will be tested for normal distribution and homogeneity of variances, and adjustments for multiple testing will also be considered.

The power calculation for the NuEva study based on a cross-sectional study which was designed to compare physiological markers and cardiovascular risk markers, such as blood lipids between habitual meat-eaters and habitual vegetarians (19). One hundred and thirty-nine healthy male subjects (vegans: *n* = 18, ovo-lacto vegetarians: *n* = 43, moderate meat eaters: *n* = 60, and high = meat eaters: *n* = 18) aged 20–55 years were recruited in Melbourne. Based on this study, meat eaters had a significantly higher cluster of cardiovascular risk factors compared with vegetarians, including increased body mass index, waist to hip ratio, plasma total cholesterol, LDL cholesterol, and triacylglycerol levels, ratio of total cholesterol/HDL and LDL/HDL, and plasma factor VII activity.

Li et al. (19) revealed a LDL/HDL ratio of 2.89 ± 0.91 in the group “high meat" (corresponding to the NuEva group: Western diet) and a significant lower LDL/HDL ratio of 2.25 ± 0.71 in the group “vegan” (corresponding to the NuEva group: vegan). Based on this data, a sample size of 44 per group had 80% power to achieve a difference of 0.6 (difference between μ1 = 2.89, μ2 = 2.25), assuming that the standard deviation is 0.9 (using a 2-side *t*-test with 0.05 as significance level; Power calculation by G^*^Power 3.1.9.2). Because of the complex study design and the long-term interval of the NuEva study, a dropout rate of 25% per group was assumed. Thus, at least 55 participants per group (n total = 220) were enrolled in the NuEva study (Figure 1).

## Discussion

Cardiovascular diseases are the leading cause of death worldwide, and the rising upward trend from 12.3 million in the year 1990 to more than 17.6 million in 2016 is alarming (20). Several risk factors contribute to an earlier onset and accelerated progression of CVD. While gender, age, and genetic predisposition are beyond control, most risk factors result from lifestyle. The Global Burden of Disease Study stated that more than 9.1 million premature deaths from CVD worldwide are attributable to dietary risks, which equals 52% of all CVD-related deaths in the year 2016 (20). The traditional Western Diet is marked by high intakes of calories, salt, saturated fat, and simple sugar, while consumption of MUFA and PUFA, whole grain fibers, fish, vegetables, fruits, vitamin D, or potassium is inadequate (21). In addition, unfavorable developments such as (i) a lack of the natural sense of appetite, hunger or satiety in combination with (ii) an oversupply of food, and (iii) the lack of knowledge about relations between food intake and health risks are marking the Western lifestyle and may contribute to the increased cardiovascular risk.

On the other hand, a reduction of animal products in the daily diet correlates with a reduction of cardiovascular risk factors. The *Dietary Approaches to Stop Hypertension (DASH)* emphasizes the consumption of fruits, vegetables, fat-free/low-fat dairy, whole grains, nuts, and legumes and limits saturated fat, cholesterol, red and processed meats, sweets, and added sugars, salt and sugar-sweetened beverages (22). This dietary pattern is associated with decreased incidence of CVD [RR, 0.80 (0.76–0.85)] and diabetes [RR, 0.82 (0.74–0.92)] in prospective cohort studies (*n* = 942,140). Besides, the implementation of the *DASH* diet improves blood pressure, total cholesterol, LDL cholesterol, HbA1c, fasting blood insulin, and body weight in controlled trials (*n* = 4,414). Thus, the *DASH diet* is widely recommended by international diabetes and heart association guidelines. Comparable results are found for the *Portfolio diet*, a plant-based dietary pattern rich in nuts, plant protein, viscous fiber and plant sterols (23).

In accordance, vegetarians have lower BMI, blood pressure, serum total cholesterol and LDL cholesterol concentrations than non-vegetarians (3). In addition, reduced rates of death from ischemic heart disease, and lower incidence of hypertension, stroke, type-2 diabetes mellitus, and certain cancers are described. Viguiliouk et al. (24) also reported that vegetarian dietary patterns improve glycemic control, LDL cholesterol, non-HDL cholesterol, and body weight (nine trials, *n* = 664). Glenn et al. (25) confirmed the favorable effects of vegetarian dietary patterns on cardio-metabolic risk factors. They included seven prospective cohort studies (197,737 participants, 8,430 events). This meta-analysis indicates, that vegetarian dietary patterns are associated with reductions in CHD mortality (~22%) and incidence (~28%) but not with CVD and stroke mortality in individuals with or without diabetes.

In summary, current data indicate an inverse association between consumption of plant-based diets and cardiovascular risk factors and CHD risk, respectively. The reduction in animal products in the daily diet is associated with a decrease in energy, fat, and particularly SFA. On the other hand, long-term implementation of plant-based dietary patterns bears the risk for inadequate intake of essential nutrients such as vitamin B12, n-3 LC-PUFA, minerals (focus: calcium) and trace elements (focus: iron, iodine, zinc, selenium). This could impair bone health and may favor the development of chronic-inflammatory symptoms or depressive symptoms, respectively (26–29).

The NuEva study is designed to identify critical nutrients for the studied diets, varying in their content of animal products and to validate the derived nutritional concepts to counteract nutrient deficiencies.

In this context, the supply of n-3 LC-PUFA and in particular, the conversion of alpha linolenic acid (C18:3, n-3, ALA) from linseed oil is also an important aspect of the NuEva study. In most countries and, particularly, in subgroups such as vegetarians or vegans, ALA-rich sources, such as walnuts, flaxseed, soybeans, and canola oils, provide most of the dietary n-3 PUFA. In humans, the enzymatic conversion of ALA into n-3 LC-PUFA is inefficient. In particular, endogenous conversion into docosahexaenoic acid (C22:6, n-3, DHA) is limited (30, 31). Plant-based diets are very low in n-3 LC-PUFA such as eicosapentaenoic acid (C20:5, n-3, EPA, <5 mg/d). The intake of DHA varies depending on consumption of DHA-enriched eggs on average <33 mg/d (32). The dominating PUFA in vegetarian/vegan diets is the n-6 PUFA linoleic acid (C18:2, n-6, LA) from seeds and oils from sunflower, soy, safflower or pumpkin. Its intake ranges from ~15 g/d (5.7% of daily energy) in omnivores to ~30 g/d (10–12% of daily energy) in vegetarian/vegan diets (33). The relatively high LA/ALA ratio in the vegetarian/vegan diet could impair the conversion efficiency from ALA to EPA and DHA (24, 25). High intake of n-3 LC-PUFA is considered to improve cardiovascular risk factors and to reduce the incidence of cardiovascular outcomes (34–39). However, the evidence for similar health-promoting effects of land-based n-3 PUFA is weak (37, 38). Thus, an adequate supply of n-3 LC-PUFA is eligible to maintain or improve health.

Adequate nutrient intake also modulates the complex process of bone mineralization and resorption. Thus, plant-based dietary regimens are traditionally considered healthy, but their real impact on bone health still needs to be examined. Plant-based diets are alkaline and tending to contain more protective nutrients, including magnesium, potassium, vitamin K, and antioxidant and anti-inflammatory phytonutrients that favor bone mineral density (BMD). The intake of dairy products as sources of calcium, high-value protein, and vitamin D may also improve bone health. On the other hand, vegetarian and vegan diets contain lower amounts of protein, vitamin D, vitamin B12, n-3 LC-PUFA, and calcium (40, 41). Moreover, the intake of oxalic acid and phytic acid, and the lower bioavailability of zinc in plant-based diets may impair the absorption and retention of calcium. Knurick et al. (42) compared BMD from young, non-obese adults consuming meat-based (*n* = 27), lacto-ovo vegetarian (*n* = 27), or vegan (*n* = 28) diets for ≥1 year. Based on 24 h diet recalls, the plant-based diets are associated with reduced protein intake by ~30% compared to omnivores. An association between dietary protein and BMD was found for those following a vegan diet. Veronese and Reginster (29) confirm that vegetarian diets (particularly vegan ones) are associated with significantly lower BMD values in comparison with meat eaters. The NuEva study aims to evaluate the impact of the studied diets on markers reflecting bone health as well as to validate the developed nutritional concepts concerning their impact on bone mineralization and resorption.

Strengths and limitations of this study:

▪ The NuEva study is designed to identify critical nutrients relating to the implementation of the studied diets (vegetarians, vegans, flexitarians, Western diet)
▪ Based on the screening data, personalized nutritional concepts are prepared to counteract under- or oversupply of essential nutrients.
▪ The personalized nutritional concepts are validated in the intervention period of the NuEva study.
▪ The design of the NuEva study provides the opportunity to identify differences in the conversion of ALA into n-3 LC-PUFA depending on the studied dietary patterns, e.g., varying in the concurrent intake of n-6 PUFA such as linoleic acid.
▪ The study allows assessing physiological benefits or possible physiological consequences resulting from the implementation of the studied nutritional habits with focus on cardiovascular risk, but also diabetes risk, inflammatory markers, bone health, thyroid function and microbiome.
▪ The lack of a control group without menu plans is a limitation of the study design.
▪ Despite the multifarious approach of the NuEva concept the individual compliance with the proposed dietary strategies stills an uncertainty factor that cannot be captured in total.
▪ The menu plans were prepared with the software PRODI® version 6.4 (Nutri-Science, Stuttgart, Germany) for professional dietary counseling and therapy. The calculations on nutrients composition based on the “Bundeslebensmittelschlüssel” and further nutrition tables^3^. In this context, limitation occur due to differences between nutrient profiles calculated with the software and the nutrient composition of the foods that are really consumed. Variations can depend on sorts, preparation conditions, feeding conditions.

## Ethics and Dissemination

▪ All human investigations are conducted according to the principles expressed in the Declaration of Helsinki. Written informed consent is a precondition for enrollment of each participant. The study protocol, including the protocols for subject recruitment, assessment, analysis of study parameters, participant information, was reviewed and approved by the Ethical Committee of the Friedrich Schiller University of Jena (number: 5504-03/18).
▪ The NuEva study was registered before launching (Trial Registration: ClinicalTrials.gov Identifier: NCT03582020).
▪ At first, a manuscript with the results of the screening period will be published in a peer-reviewed journal. Secondly, separate manuscripts will be written on the results of the intervention period, addressing each focus of the NuEva study such as cardiovascular risk, diabetes risk, inflammatory markers, bone health, thyroid function and microbiome, but also the conversion of land-based n-3 PUFA into their long-chain metabolites. These manuscripts will also be submitted for publication in peer-reviewed journals.

## Data Availability Statement

The article includes the description of a human trial (protocol). No datasets are presented in this article. Requests to access results or datasets should be directed to the corresponding author.

## Ethics Statement

All human investigations are conducted according to the principles expressed in the Declaration of Helsinki. Written informed consent was a precondition for enrollment of each participant. The study protocol, including the protocols for subject recruitment, assessment, analysis of study parameters, participant information, was reviewed and approved by the Ethical Committee of the Friedrich Schiller University of Jena (number: 5504-03/18).

## Author Contributions

CD planned and performed the NuEva study (inclusive recruiting and conduction of the nutritional counseling units as part of the NuEva concept). She was responsible for preparation of the used information materials and personalized menu plans, sample collection, management of the study data, analysis of the study parameters, and their statistical evaluation. CD wrote the paper and approved the final version of the manuscript.

## Conflict of Interest

The author declares that the research was conducted in the absence of any commercial or financial relationships that could be construed as a potential conflict of interest.

## Footnotes

^1^Available online at: https://vebu.de/veggie-fakten/entwicklung-in-zahlen/vegan-trend-fakten-zum-veggie-+/

^2^Available online at: https://www.bmel.de/SharedDocs/Downloads/Ernaehrung/NVS_ErgebnisberichtTeil2.pdf?__blob=publicationFile

^4^Available online at: https://www.nutri-science.de/software/prodi.php
